# Ageing in Place Classification: Creating a geodemographic classification for the ageing population in England

**DOI:** 10.1007/s12061-022-09490-y

**Published:** 2022-12-12

**Authors:** Yuanxuan Yang, Les Dolega, Fran Darlington-Pollock

**Affiliations:** 1 University of Liverpool, Liverpool, UK; 2 University of Leeds, Leeds, UK; 3 University of York, Heslington, UK

**Keywords:** Geodemographics, Area classification, Ageing, Population

## Abstract

Population ageing is one of the most significant demographic changes underway in many countries. Far from being a homogenous group, older people and their experiences of ageing are diverse. A better understanding of the characteristics and geography of the older population, including the older workforce, is important. It allows policymakers and stakeholders to better adapt to the opportunities and challenges that the ageing population brings. This paper describes the implementation of the Ageing in Place Classification (AiPC) in England. AiPC is a multidimensional geodemographic classification, and it employs a wide range of spatially representative attributes of older people’s sociodemographic characteristics and their living environment at the small area level. The openly available product provides valuable insights that can be implemented in both local and national contexts, in particular to improve service delivery and inform targeted policy interventions. AiPC is readily updateable with the arrival of new Census data; the concept and framework are also transferable to other countries.

## Introduction

The population of England is ageing. By 2041, approximately 26% of the UK’s population will be aged 65 and over, with ages 50 and over likely comprising around half the adult population (ONS, 2018). Concurrently, the success of the economy will increasingly be linked to an ageing workforce: the proportion of workers aged between 50 and State Pension Age (SPA) is projected to increase by 9% to 35% over the next 30 years (Government Office for Science, 2016). This dramatically shifting age-structure challenges the fiscal sustainability of already strained models of service provision. Indeed, the Lords Economic Affairs Committee has condemned the current provision of social care for vulnerable older people (Butler, 2019), while the Department for Business, Energy and Industrial Strategy (2019) counts an ageing society as one of the four Industrial Strategy Grand Challenges. It is therefore imperative to develop a robust evidence base that can support effective planning and policy intervention. As the social, economic and environmental requirements of an older population (individual- and population-level) will be significantly different from those previously encountered (RTPI, 2004: 2), effective planning and policy intervention will follow from a better understanding of the nature and geography of the older population. However, while population ageing is often demonised as a looming crisis wherein older people are homogenised as a dependent burden, the characteristics, behaviours and needs of the older demographic are not uniform, tending to vary spatially (Skinner et al., 2014). Challenging binary divisions between the young (able) and old (infirm) is therefore critical, both in the overall population and within the workforce.

To better understand the social and spatial heterogeneity within the older population and thereby support effective policy development and targeted service provision, this project develops an open access, multidimensional classification of the older population in England at a small area level. Geodemographics, built with cross-sectional data, are widely used to support service planning and policy development (e.g. the Output Area Classification built with and used by the Office for National Statistics). Though a cross-sectional snapshot, such classifications can provide valuable insights into the nature of need, vulnerability and opportunity in a population which will continue to age. This understanding provides a robust basis for effective planning and policy intervention.

The paper proceeds in the following way. First, it provides an overview of literature related to various geodemographic classifications and the rationale for creating a bespoke geodemographic classification of ageing population. Second, it outlines the data, computation of variables and methods employed to create the Ageing in Place Classification. Then it presents the results including the characteristics of the derived clusters and finally, it discusses the findings and their implications within the context of how such national classification can enhance our understanding of the needs and support local service provision for older generations.

## Need for a Geodemographic Classification of Ageing Population

Emerging policy agendas prioritising *ageing-in-place* (Bartlett & Carrol, 2011) and calls for employment reforms for age-adjusted and flexible working patterns and age-appropriate healthcare for older workers (see Ilmarinen, 2006) are contributing to the realisation of age-friendly societies. In the UK, an age friendly society is defined as ‘a place where people of all ages are able to live health and active later lives… [where it is] possible for people to continue to stay living in their homes, participate in the activities that they value, and contribute to communities, for as long as possible’ (see Centre for Ageing Better, n.d.) Associated policies therefore typically promote healthy, active lifestyles alongside sustained age-appropriate economic activity while also enabling older populations to live within the community for longer, rather than moving away to residential care. Their success hinges on appropriate local service provision responding to local needs, and appropriate housing. Understanding the characteristics and geography of the older population, including the older workforce, will substantively enhance policy-makers ability to meaningfully tailor and target specialised policy interventions. Such policies which then prioritise *ageing in place*, where age is not considered a barrier, are seen as advantageous in relation to individual’s sense of attachment, connection and feelings of security and familiarity to both homes and communities (Wiles et al., 2012).

Effective planning for ‘whole life-course’ neighbourhoods therefore depends on recognition of the heterogeneity of the older population and their uneven spatial distribution. Carefully targeted interventions in local service provision and the built environment (e.g. housing) will be incumbent upon a fine-grained understanding of the dynamics of place-based ageing, yet such understanding is currently lacking. Singleton and Spielman (2014) demonstrate that the social and spatial heterogeneity of population or a particular group of people can be facilitated by using a multidimensional geodemographic classification.

Geodemographic classification organises areas into categories sharing similar attributes across multidimensional variable space (Singleton & Spielman, 2014). It incorporates “geo”, implying the place and environment where people live, with “demographics”, indicating the various sociodemographic characteristics of households or individuals (Leventhal, 2016; Xiang et al., 2018). There are various geodemographic classifications worldwide, and broadly, they can be classified as either general-purpose or bespoke (e.g. education-specific) classifications (Cockings et al., 2020). Some examples of general-purpose geodemographic classification include the UK’s Output Area Classification (OAC) and P2 People and Place Classification, and the Mosaic and CAMEO classification in several countries (e.g. UK, US, Australia, Canada) (Gray et al., 2021; Singleton & Spielman, 2014). There are also new attempts and machine learning model innovations in recent studies; for example, De Sabbata and Liu (2019) incorporated autoencoders and geographic convolution in neural networks to create a geodemographic classification based on UK Census data, and the results reached high cluster homogeneity. These classifications normally cover the general population and their characteristics in local areas, and are designed for use across different applications. Despite capturing a range of important and general features, their generalist approach means they cannot always offer rich insights into all particular groups of interest (Gray et al., 2021).

Bespoke classifications, however, have been developed to support specific applications or focus on a particular group of people. These classifications rely on domain understandings and suitable data sources (Gray et al., 2021). Examples include the Internet User Classification in the UK (Singleton et al., 2020) or the mortality risk classification of Cyprus (Lamnisos et al., 2019). They are able to shed light on the diversity and inequalities in different domains and areas, such as education inequality in Beijing (Xiang et al., 2018), and in UK higher education (Singleton & Longley, 2009). Elsewhere, Benbrahim Ansari (2021) utilised unsupervised deep learning with self-organising maps for creating a geo-marketing segmentation for Business to Business industrial automation market, it allows users to better identify market demands. A local fuzzy geographically weighted clustering model was applied in the work of Grekousis (2021) to generate the geo-segments of cancer incident. The similarity approach is also adopted for understanding the inequalities in COVID-19 deaths (Grekousis et al., 2021), which is closely related to the aged population due to the higher death rate among older people. Many bespoke classifications used novel data from a range of sources, not limited to typically used Census data, to enrich and deepen insights. For example, online purchase records, internet speed data and individual survey data are used in the work of Singleton et al. (2020); and Xiang et al. (2018) has incorporated school enrolment data into the classification.

Currently, there are very few bespoke geodemographic classifications related to older people, although ageing population is of increasing importance in many countries. The closest attempt is the work of Hunter (2016), which focuses on creating an older people geodemographic classification in Australia. However, the model in this study (Hunter, 2016) was constructed purely based on the Australian Census data, rather than drawing on complementary alternative, novel sources.

The sustained marginalisation of older people in policy and public rhetoric, laid bare through the ageist discourses permeating policy and debate through the COVID19 pandemic (Darlington-Pollock et al., 2020; Ehni & Wahl 2020; Rahman & Jahan 2020), have increased the urgency to develop new, bespoke classifications of older people. Blanket advice to shield based on arbitrary age thresholds rather than more empirical evidence of need, for example, illustrate the importance of a more nuanced understanding of the heterogeneity in our older population. A bespoke classification of older people in England would facilitate this.

Developing a fine-grained understanding of the characteristics and geography of England’s ageing population at a small area level is critical to better understand the needs of these diverse demographics. It will facilitate efficient service planning and policy development, ensuring services are targeted to those most in need rather than on assumptions couched in age. For example, increasing age is associated with increasing risk of loneliness. This may prompt the targeting of befriending serves in areas according to percentage of the populated aged over a particular threshold. Yet other factors are important in shaping risk of loneliness, including marital status, levels of social activity, self-assessed general health and mental wellbeing (Dahlberg et al., 2022). Targeting befriending services because of age alone may miss those most in need while the existence of a bespoke classification of older people would mitigate against that.

## Methods and Analysis

### The Overarching Method

**Fig. 1:**
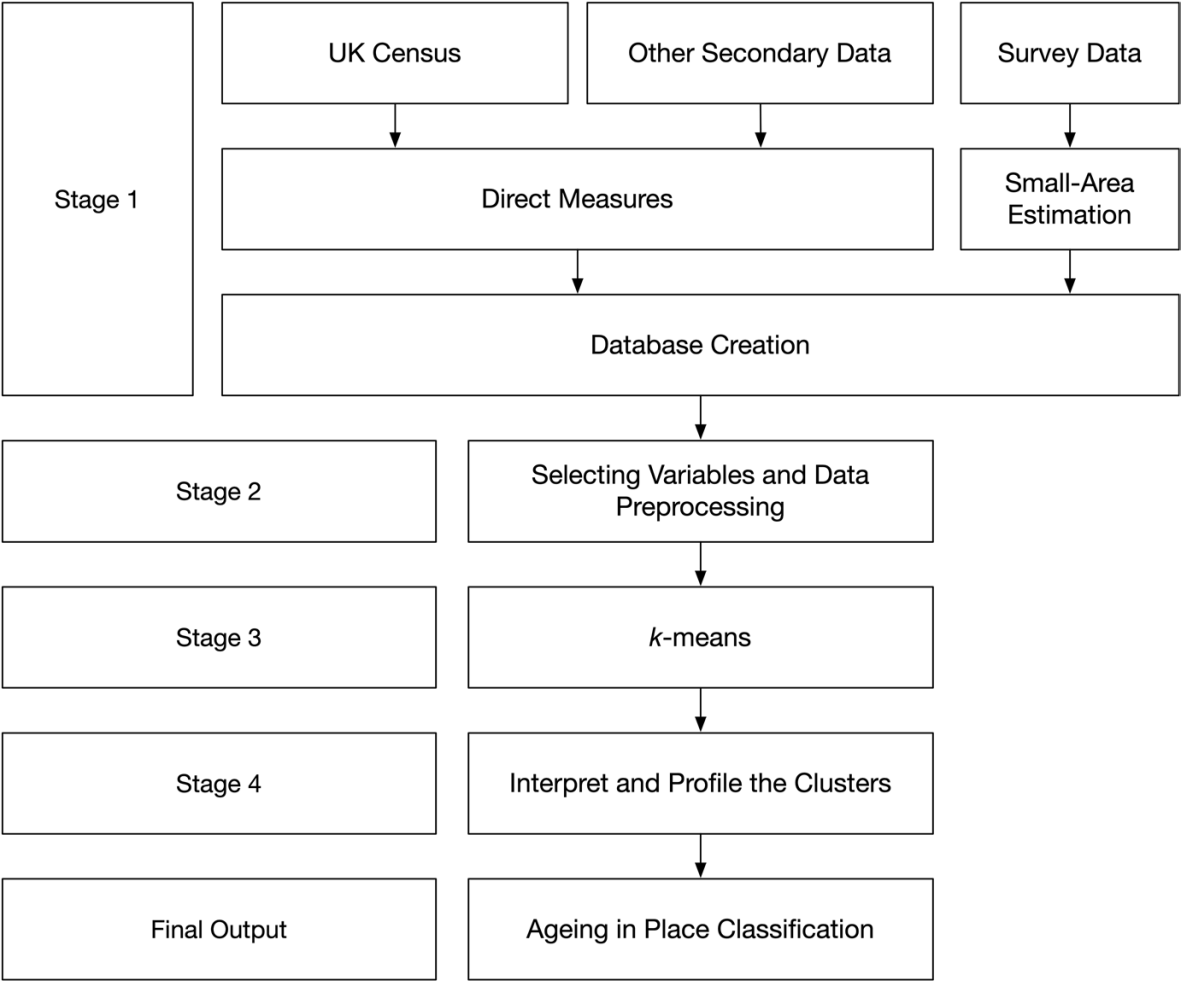
Methodology flow chart of AiPC

The overarching method of creating AiPC is similar to the approaches that have been used in other opensource geodemographic classification studies, such as the Output Area Classification (Gale et al., 2016), Classification of Workplace Zones (Cockings et al., 2020) and the Internet User Classification (Singleton et al., 2020). Our approach involved the following key steps below (also shown in Fig. 1):

1. Firstly, key domains related to older people and the places in which they live were identified based on a review of relevant literature, and validated through discussion with an expert advisory panel (these were later validated using a groundtruthing exercise). An initial set of variables and metrics were then generated from various sources to reflect different characteristics of the domains identified. Variables include direct measures that are obtained from the UK Census and other measures derived from secondary data such as NHS English prescribing data and the British Population Survey.
2. Statistical analysis of the initial set of variables and metrics including examination of their distribution, spatial coverage and patterning, as well as correlation between variables. Sensitivity analysis was also performed to measure the impact of variables on the cluster forming process (Gale et al., 2016). The statistical evaluation informed dialogue with the stakeholder and advisory group leading to the selection and reduction of the final set employed in the classification.
3. The final set of input measures obtained and assembled were normalised and standardised to ensure that all contributed equally to the clustering process (Gale et al., 2016). The unsupervised learning model of *k*-means is applied to generate a series of clusters and nested sub-clusters that represent the grouping of small areas with similar characteristics of older people and their living environment.
4. The unique characteristics of all clusters and sub-clusters were then obtained and their profiles—the so-called “Pen portraits” – created to provide further details about their diverse attributes and spatial patterns. Finally, the variables were mapped to help the end-users to understand and utilise the classification product more readily.

Several advisory group meetings and consultations were conducted to ensure the various choices in the above steps were methodologically robust and theoretically grounded. Central to the consultation exercise was identifying variables that best captured relevant characteristics of older people and the places they live, pertinent to their experience of old age and ageing. The rest of this section (Methods and analysis) provides more details of some key methods and models used to create the AiPC.

### Relevant Domains

The research focuses on creating a geodemographic classification of older people (aged 50 and over) in England at the small area level with a general aim to enable a better understanding of the geography of this demographic. Such a classification would allow for more effective service planning and policy development in the face of an aged and ageing population.

To inform the selection of appropriate variables to include, we considered two established frameworks relating to the experiences of aged and ageing people, and the places in which they live and work: (1) World Health Organization (WHO) Age-Friendly Cities Framework (AFCF), and (2) WHO Active Ageing Policy Framework (AAPF). Review of these frameworks signalled key aspects to be represented in the domains of our AiPC, respectively spanning areas such as social infrastructure, physical infrastructure, social capital, the physical environment and aspects related to the socio-economic and demographic charactristics of older people themselves.

**Fig. 2:**
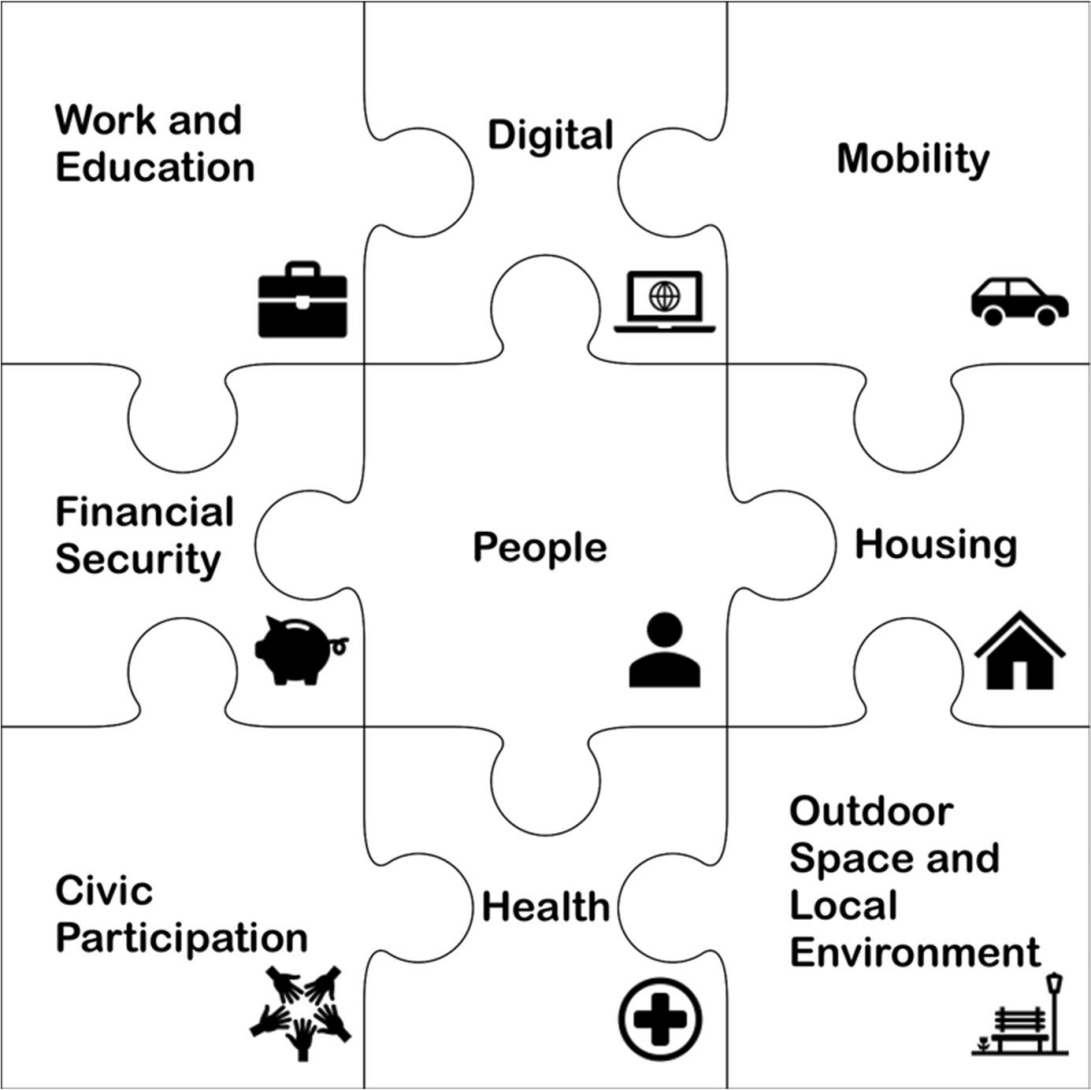
Domains of the classification of older people in England

Review of these established frameworks, relevant literature and consultation with our expert advisory group allowed us to identify nine inter-connected AiPC domains, reflecting the characteristics of older people and the places in which they live (Fig. 2). These are (1) People; (2) Housing; (3) Work and Education; (4) Mobility; (5) Financial Security; (6) Digital; (7) Health; (8) Outdoor space and living environment; and (9) Civic participation (Fig. 2). A key feature of the consultation process underpinning the development of the AiPC was the ability to ensure the final classification reflected key concerns of the end users and that it is useful in developing policy interventions and guiding decision-making.

Following decisions as to the domains of the AiPC, an initial set of variables and metrics were identified, gathered, generated and reviewed as potential inputs, with the majority of the variables sourced from the 2011 Census.

An advantage of the UK Census dataset is the availability of the Local Characteristics (LC) tables. LC provides detailed results that cross-tabulate two or more topics/dimensions, showing the greatest level of detail. The age dimension is available in many LC tables; therefore, it is possible to derive information focusing on only older people using a threshold of 50 years old or other age-adjust methods. This differentiates our classification from other extant geodemographic classifications (e.g. Output Area Classification (Gale et al., 2016)), where the total (or total adult) resident population is used as the variable denominator. In contrast, AiPC uses the number of older people in each LSOA (Lower Layer Super Output Areas, a geography designed to have a mean population of 1500) as the denominator where possible in the Census. Using the total adult population or total population as the denominator would mask key patterns and nuances pertinent to our efforts to unpack the spatial and social heterogeneity of the older population. LSOA units have the merit of keeping a good balance between fine spatial granularity and robustness in result estimation – they are therefore routinely used by policy makers (Green, et al., 2018). LSOA was also utilised in a number of established geodemographic classifications or rank measures in England, such as the Internet User Classification (Singleton et al., 2020), the English Index of Multiple Deprivation, and the Access to Healthy Assets and Hazards (Green, et al., 2018).

A number of other secondary data sources are also used to capture the additional characteristics in the above domains. These provide enhanced and complementary information relating to the older people, and their experience of ageing, to that offered by the Census. Most secondary datasets are available at LSOA level; for those not aggregated at LSOA geography, small area estimation and unit transformation techniques are used to provide LSOA estimates. “Calculating dementia treatment prescribing rate at LSOA level”, “Estimating digital engagement of older people” and “Deriving measures from other secondary datasets” sections below introduce key complementary variables used to create AiPC and the methods to compute them, whilst the full list of domains and all corresponding variables are shown in the Appendix 1.

### Calculating Dementia Treatment Prescribing Rate at LSOA Level

Dementia impacts older people’s cognitive, functional ability and behaviours, this may lead to a compromised involvement in activities, social networks and society, possibly resulting in decreased quality of life (van Corven et al., 2021; Wittenberg et al., 2019). The AiPC have included two variables in the Health domain to describe the spatial variations of dementia treatment at LSOA level: (1) Prescribing rate of Acetylcholinesterase inhibitors in each LSOA per person (50 +) per year; and (2) Prescribing rate of Memantine in each LSOA per person (50 +) per year.

English Prescribing data from January 2015 to December 2019 were used to characterise the prescribing pattern of dementia treatment medication over LSOAs in England. Monthly prescribing data were obtained from the NHS Business Services Authority.^1^ The data contains records of the prescriptions issued by each GP practice, including the ID of the practice, the prescription BNF code, Chemical substance and quantity (the number of items prescribed), and the year and month of the prescription. However, the information about individual patients of each prescription is not available, and therefore it is not possible to infer the age of the patients.

**Fig. 3:**
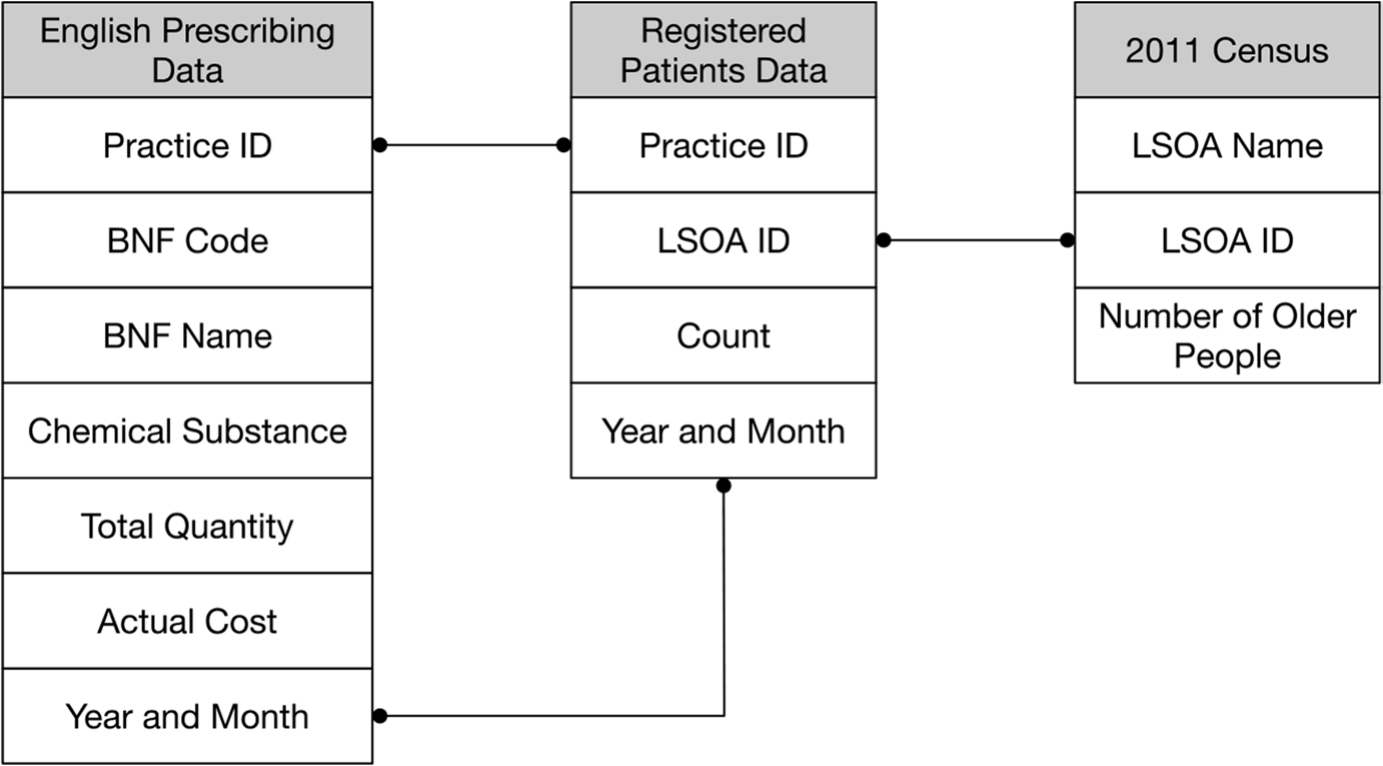
The relations between NHS English prescribing data, registered patients data and 2011 census

To estimate the prescribing rate at LSOA level, the counts of patients and their home address LSOA registered at each GP were obtained from the NHS Digital Website.^2^ The two datasets and the 2011 Census were then linked by practice ID, Year and Month and LSOA ID, as shown in Fig. 3.

Two main types of medicines are used for dementia treatment, and they are Acetylcholinesterase inhibitors and Memantine. More specifically, Memantine is usually used for moderate to severe dementia, and its prescribing rate helps to reveal the spatial variations of more advanced dementia treatment at the small area level.

This work used an aggregation unit transformation approach suggested by Comber et al. (2021). The steps involve:

1. The dementia medication prescription records were extracted based on the chemical substance.
2. The number of dementia medication items prescribed by each GP practice each month was determined.
3. The proportion of patients registered at each GP practice in each LSOA was calculated.
4. These were used to allocate prescriptions to LSOAs based on the proportions of patients from each GP practice.
5. The prescribing rate (items per older person per year) for each LSOA was calculated using the LOSA older population from the Census 2011.

It should be noted that dementia is highly associated with age, but this work does not produce age-standardised dementia medication prescribing rates due to data limitations. Therefore, the result can only indicate places with higher levels of prescribing for dementia medications. These are not adjusted by age. A higher prescribing rate should not be interpreted as a higher prevalence of dementia in a particular area. Rather, it is indicative of the treatment environment in which older people, themselves at higher risk of being diagnosed with dementia, live in. When combined with local understanding and knowledge of other dementia-risk factors in an area and the health service infrastructure, this can help end-users of this classification better understand the possibility of need and importantly, unmet need, in their local population.

### Estimating Digital Engagement of Older People

Digital is one of the key domains in the AiPC. In an increasingly digitised world, where the internet not only provides quick access to information but increasingly access to a range of services, access to the web in older age may be an important component to well-being in later life. Indeed, it is a potential tool to reduce social exclusion (Szabo et al., 2019), where properly accessible as the recent experiences of national lockdowns in the COVID19 pandemic made clear. But issues of access and engagement remain. There are no data that can be used readily to measure older people’s internet engagement in England at a small area level. Hence, this study adopted a novel Small Area Estimation technique that leverages Spatial Microsimulation (SMS) and a Machine learning model, applying the method used to create the Internet User Classification (IUC) (Singleton et al., 2020). Building on their approach, we use the British Population Survey (BPS), which contains information about people’s sociodemographic characteristics, internet access and engagement. Only data for those aged over 50 in England was selected and used in the following modelling framework.

The individual-level BPS data were firstly combined with Census local characteristics data to build an SMS model, implementing the Iterative Proportional Fitting Procedure (IPFP) with the constraint variables that are listed in Table 1. IPFP is an iterative procedure that is normally applied with the SAE microsimulation framework to reweight individual sample data (BPS in this case) for each small area (LSOA) to match benchmarks (Census) (Lomax & Norman, 2016; Lovelace et al., 2014; Singleton et al., 2020). The SMS results can then be used to calculate the variables of interest, which are the percentage of older people in the LSOA who have broadband access at home, use the internet for information seeking, use the internet for online shopping and banking, and use the internet for social purposes. These variables were selected in consultation with our stakeholder and expert advisory group.

**Table 1:** Common variables used as SMS constraints

Variable	Categories
Age	50–54; 55–59; 60–64; 65–69; 70–74; 75–79; 80 and above
Gender	Male, Female
Qualification	No qualification or other qualification; Level 1, 2 or apprenticeship; Level 3,4, or higher
Social Grade	A and B; C1, C2, D and E
Economic Activity	Part Time; Full Time; Self Employed; Unemployed; Retired; Look after family; Long Disabled; Student (Working and Not working); Not Working Other

The estimated percentage values from SMS are then used to train a series of XGBoost models, which is a tree-based supervised machine learning model. To build a robust model, only LSOAs with 11 or more observations were used for training purposes, which accounted for 3198 LSOAs (9.74% of total LSOAs). These were further sample weighted by the number of observations.

### Deriving Measures from Other Secondary Datasets

In addition to Census, NHS data and BPS, a number of other secondary datasets are also used in this study to provide further direct measures related to older people and their living environment. They are distributed across different domains, and the key ones are introduced here. Full details about these data and the derived measures are provided in the acknowledgement and Appendix 1.

The Access to Health Assets and Hazards (AHAH) dataset and the Journey Time Statistics (JTS) were used to describe (physical) access to different services and locations, air quality and green space. Index of Multiple Deprivation (IMD) scores were used to obtain information on older people’s income deprivation, housing quality and the context of local crime rates. Furthermore, a median house price indicator derived from ONS HPSSA (Mean house prices by middle layer super output area) dataset was also used to build the AiPC. Finally, we used the Ordnance Survey POI (points of interests) data to estimate the capacity for civic activity as a proxy for civic participation. This was calculated as a number of civic assets in a buffer zone (1 km) of each LSOA, divided by the number of residents in each LSOA.

### Selecting Variables and Pre-Processing

Variable selection is a crucial phase of developing a geodemographic classification, ensuring the development of meaningful and application-relevant clusters. (Liu et al., 2019; Murphy & Smith, 2014). It is important to find a balance between “the fewer variables, the better” approach (Openshaw, 1995) and the utility and coverage of domains and features. Therefore, a series of variable evaluations were performed, which assisted with selection of variables that (1) fell within the scope of the classification; (2) were of good quality and representativeness; (3) varied the most between areas, thus offering the most discrimination potential (Singleton & Longley, 2015); and (4) were not strongly correlated with each other to avoid unnecessary weight in a particular dimension in the classification(Cockings et al., 2020; Gale et al., 2016; Singleton & Longley, 2015).

First, the mean, median, quantile values, standard deviation, skewness index, histograms, normal probability plots, maps at various geographical scales and a correlation matrix were examined (Cockings et al., 2020). Then cluster-based sensitivity analysis was conducted to identify variables that had the greatest impact, either positive or negative, on cluster formation (Liu et al., 2019). This involved assessing the Silhouette, Calinski-Harabazs and Davies-Bouldin scores after excluding different variables from a cluster run. Those with negative impacts were carefully examined, leading to, for example, decisions to either exclude or combine those with other variables (Gale et al., 2016; Liu et al., 2019).

An initial set of several hundred metrics was first reduced to 121 variables, and a final set of 71 variables, covering all the nine domains as listed in Fig 2. The final set of variables is provided in Appendix 1. Before running the clustering models, the input variables were pre-processed to reduce some undesirable impact in their raw format (Cockings et al., 2020; Gale et al., 2016). This included normalisation and standardisation to “transform the variable values to approximate normal distribution” (Brunsdon & Singleton 2015), ensuring an effective cluster formation can be achieved. Different normalisation methods were tested in this study, including Log, Box-Cox and Inverse Hyperbolic Sine transformations. Similar to the work of Cockings et al. (2020), the investigation has identified Box-Cox as returning the best performance in reducing the skewness of variables. Therefore, Box-Cox transformation was chosen to normalise the variables. Range-scale was then used for the standardisation purpose which creates a universal scale of the measurement for every variable (Brunsdon & Singleton; Xiang et al., 2018).

### Clustering Analysis

In clustering analysis, different algorithms can be used to obtain grouping solutions and to then create the final geodemographic classification. For example, PAM (Partition Around Medoids) clustering (Brunsdon et al., 2018), geographic convolutional neural networks (De Sbbata & Liu 2019), and local fuzzy geographically weighted clustering model (Grekousis, 2021). Before determining which clustering model to use, some models (*k*-means, PAM, and sample weighted *k*-means) were tested, followed by consultations with expert stakeholders. For AiPC, the end users (stakeholder community) expressed their preference for keeping the model relatively consistent with established open geodemographics classification products in the UK. As a data product, this helps to ensure the AiPC results are more intuitive for users and can be comparable to other products, such as the OAC (Gale et al., 2016) or COWZ-UK (Cockings et al., 2020). Therefore, we use a top-down *k*-means clustering model.

The *k*-means algorithm was repeatedly applied to obtain a two-tier hierarchy structure of classification (Cockings et al., 2020). The top-level of AiPC, referred to as *supergroups*, was first determined, and then each cluster in the first tier was examined and further divided to generate the second-tier results, referred to as *groups*.

**Fig. 4:**
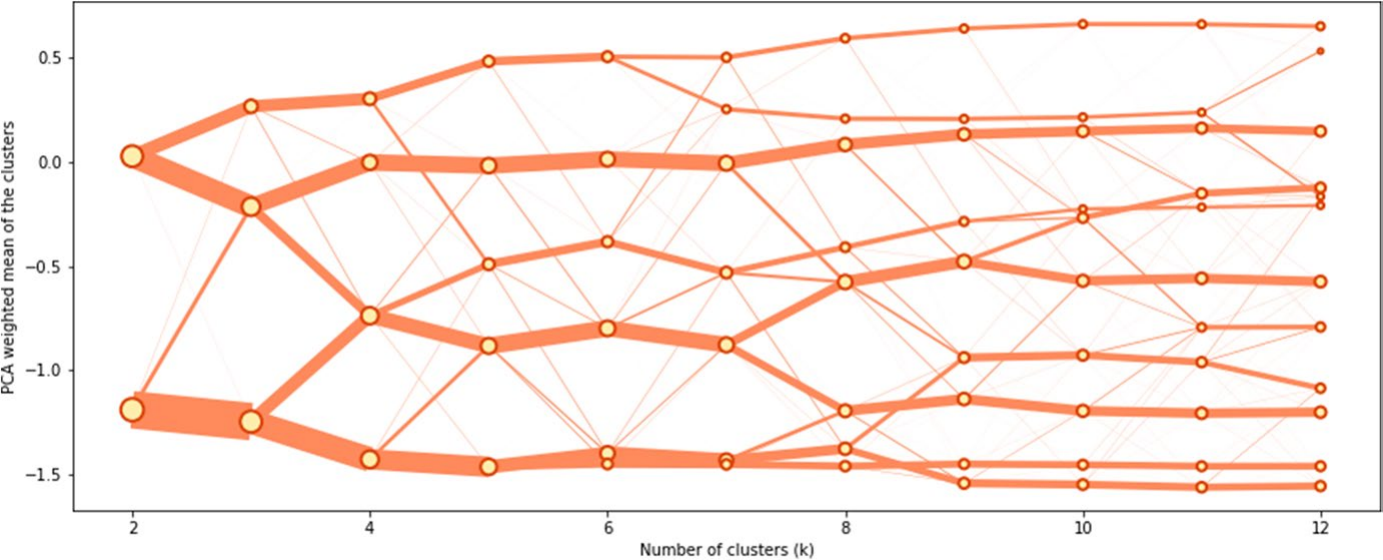
Clustergram of the first tier classification

Clustergrams were used to determine the most suitable number of clusters in each tier (Fig. 4). This visual tool plots different potential *k* values with the weighted mean of their first principal components (Schonlau, 2002). The X-axis indicates the different number of clusters, and y-axis represents the input variable space. Each point reflects the centre of a cluster, weighted by its first principal component. The lines between points shows how new clusters are formed from the splitting and merging in different segmentation. Line thickness represents the number of samples moving between clusters (Schonlau, 2002).

Clustergram is able to demonstrate at which point (number of *k*), the clusters are well separated in the input variable space (the y axis), so the suitable number of clusters can be identified (Cockings et al., 2020; Singleton et al., 2020). The clustergram for the top level of the AiPC hierarchy, shown in Fig. 4, suggested that, *k* = *5* is most appropriate – all the clusters are well separated along the y axis.

Each of the identified first-tier clusters was then subset and again examined using clustergram to determine the number of sub-clusters in the second-tier.

### Naming and Describing Clusters

The classification results, obtained by using the steps above, were assigned a first-tier classification code, and a second-tier code. The supergroups (first-tier) and groups (second-tier) were given a name and descriptions for each cluster, referred to as “Pen Portraits” based on their principal and distinctive variables (Gale et al., 2016; Xiang et al., 2018). The principal features of each supergroup and group were summarised using their mean z-score across all the input features and illustrated with radar plots and bar plots. The z-score value of 0 denoted the England’s average, while higher values indicate higher than average and vice-versa. The highest and lowest z-scores computed for each variable are helpful in providing the key and principal feature of the cluster and contributed to providing cluster profiles.

This process, especially the naming, is challenging and often contentious (Gale et al., 2016; Vickers & Rees, 2011). The names strive to (1) accurately reflect the input variables; (2) be consistent throughout the hierarchy; (3) remain neutral; and (4) avoid duplicating names/labels with other classifications (in the UK, for example, OAC) (Cockings et al., 2020). To maximise the utility of the names and pen portraits for end-users of this classification, the creation of the pen portraits was done in consultation with the expert advisory group and a ground-truthing exercise. Names and pen portraits were first proposed by the authors, and then evaluated through a ground-truthing exercise and advisory board consultation (Vickers & Rees, 2011). This process also helps collect further feedback and suggestions, and the names and descriptions are then revised accordingly.

## Results: The Ageing in Place Classification (AiPC)

### The Structure of AiPC

**Fig. 5:**
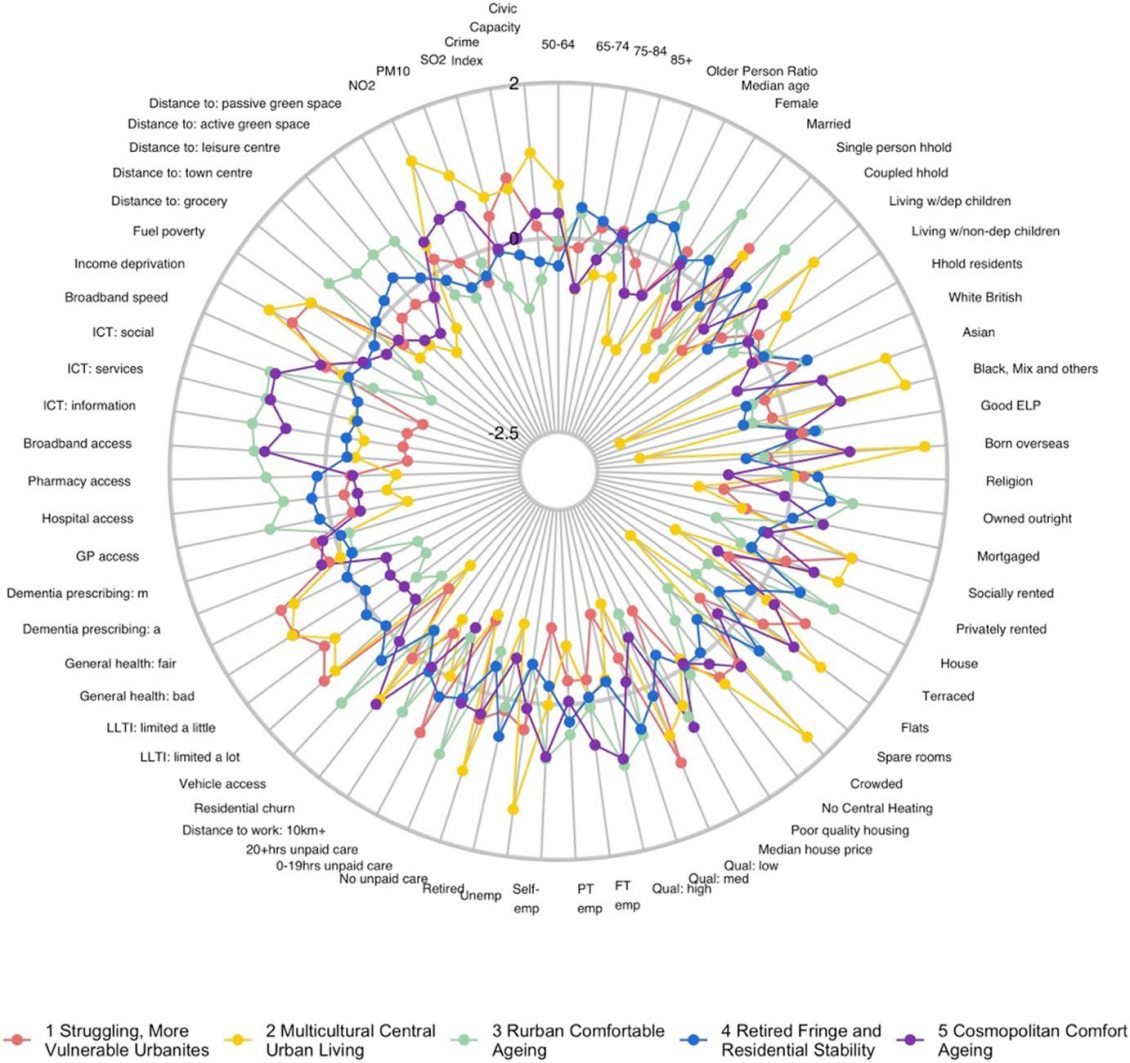
Mean z-scores, supergroups

The AiPC consists of two tiers (Table 2). The top-level supergroups (tier 1) of AiPC, contains five clusters providing the most generic descriptions of the older population and their living environments (see Fig. 5). Tier 2, the nested groups, contains 13 subclusters and further differentiates the characteristics of the older population within the five supergroups of tier 1.

**Table 2:** AiPC hierarchy and cluster names

Supergroups	Groups
1. Struggling, More Vulnerable Urbanites	1.1 Disadvantaged Single Households
	1.2 Struggling White British
	1.3 Terraced Mix, Relative Stability
2. Multicultural Central Urban Living	2.1 Inner City Diverse Living
	2.2 Peripheral Constrained Diverse Living
3. Rurban Comfortable Ageing	3.1 Rural Comfortable Ageing
	3.2 Ageing in the Affluent Fringe
4. Retired Fringe and Residential Stability	4.1 Retired Country and Coastal Living
	4.2 Comfortable Rural/Suburban Ageing Workers and Retirees
	4.3 Constrained Semi-Rural Ageing and Retirement
5. Cosmopolitan Comfort Ageing	5.1 Cosmopolitan Family Ageing
	5.2 Coastal Later Aged Retirees
	5.3 Cosmopolitan Ageing

The Pen Portraits (Sections “Supergroup 1: Struggling, More Vulnerable Urbanites”, “Supergroup 2: Multicultural Central Urban Living”, “Supergroup 3: Rurban Comfortable Ageing”, “Supergroup 4: Retired Fringe and Residential Stability” and “Supergroup 5: Cosmopolitan Comfort Ageing”) of the supergroups (tier 1), unless otherwise stated, compare each characteristic (variable) to the “average” characteristics for England as a whole (the global mean), however, the group’s (tier 2) mean values are relative to relevant mean input values of the parent group. Table 3 summarises the size, age, and age-structure of each of the five supergroups, where the Older Person Ratio is defined as the population aged 65 and over, relative to the population 18–64 in each LSOA. Overall, there are three larger and two smaller clusters with Supergroup 3, ‘Rurban Comfortable Ageing comprising 8,802 LSOAs, 32.6% of the total population of England aged 50 and over, and with a median overall age of 45.37 being the largest and oldest cluster (Table 3). Conversely, Supergroup 2, ‘Multicultural Central Urban Living’ is the smallest and youngest cluster, comprising 3,905 LSOAs, only 7.7% of the total population aged 50 and over, and with an overall median age of 30.50. This cluster is also the most ethnically diverse, reflecting the relative youth of the ethnic minority population of England.

**Table 3:** Supergroup summary characteristics – size, age, and age-structure

Code	Supergroup	Number of LSOAs	Percentage of England’s population aged 50 +	Mean Median Age	Mean Older Person Ratio
1	Struggling, More Vulnerable Urbanites	7,507	20.20%	36.25	0.25
2	Multicultural Central Urban Living	3,905	7.70%	30.5	0.13
3	Rurban Comfortable Ageing	8,802	32.60%	45.37	0.35
4	Retired Fringe and Residential Stability	8,194	27.90%	43.2	0.34
5	Cosmopolitan Comfort Ageing	4,436	11.50%	36.11	0.2

**Fig. 6:**
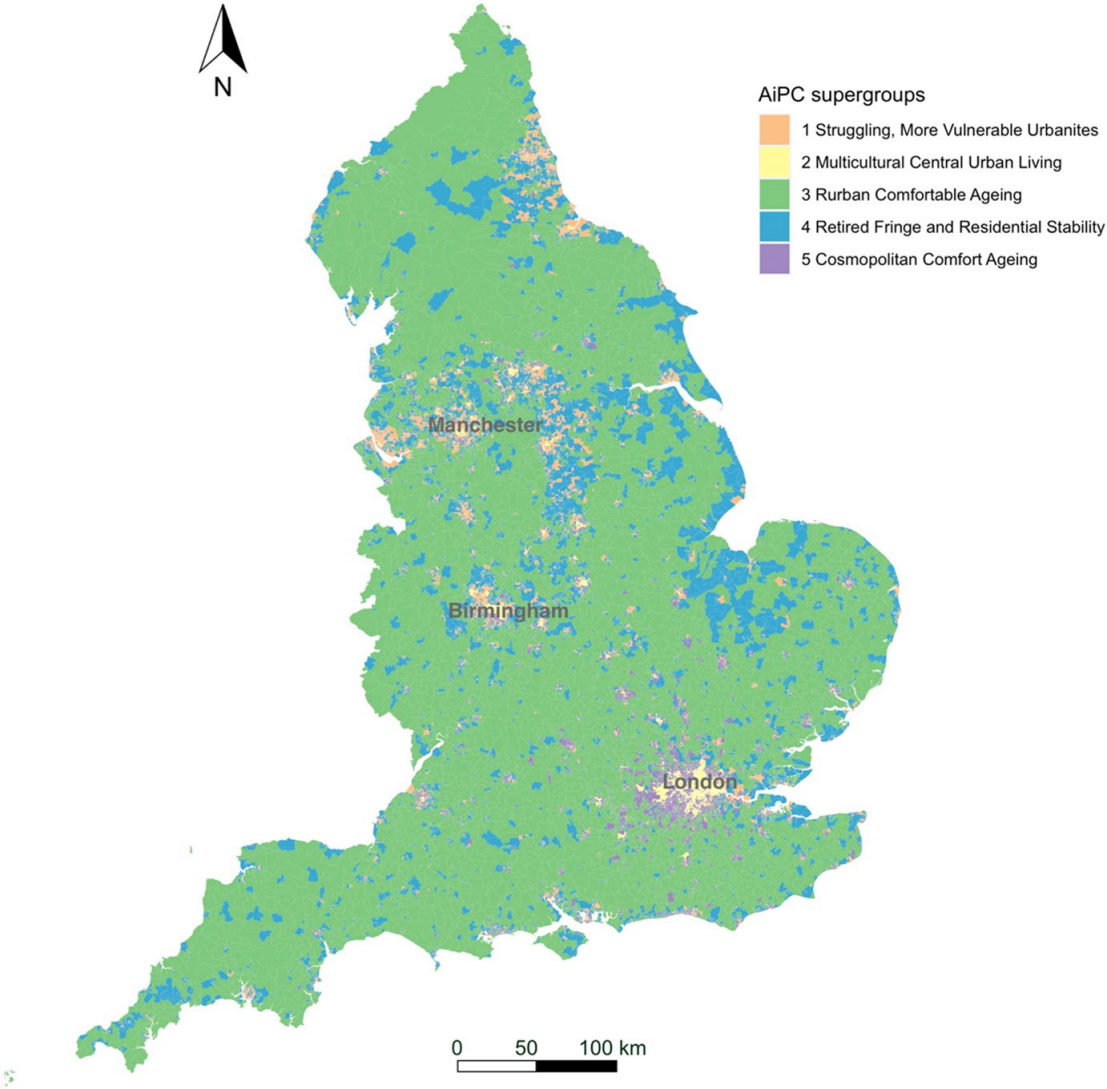
Map of AiPC supergroups (first tier)

**Fig. 7:**
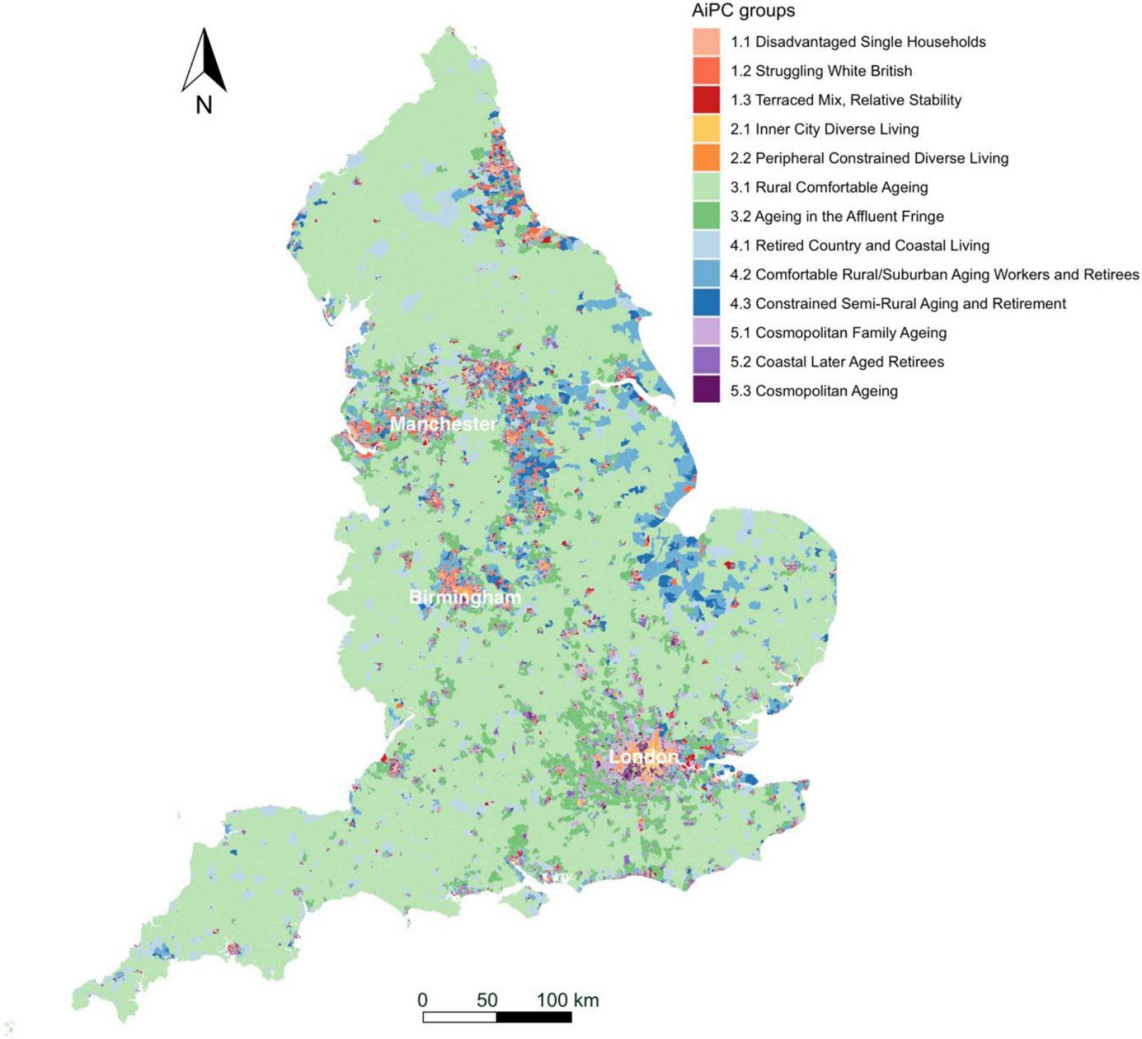
Map of AiPC groups (second tier)

The five supergroups are described in the next section (Section “AiPC pen-portraits”), including their names and distinctive characteristics. More details and statistics on the groups within each supergroup are provided in Appendix 2 (Figs. 6 and 7).

### AiPC Pen-Portraits

#### Supergroup 1: Struggling, More Vulnerable Urbanites

**Fig. 8:**
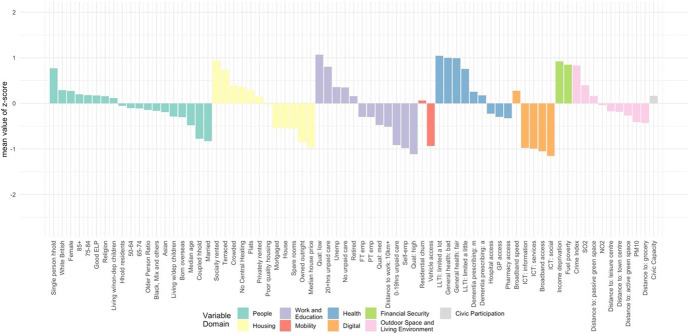
Profiles of supergroup “Struggling, More Vulnerable Urbanites” (Mean z-scores). *Note: Variables ranked within a domain by mean z-score. All future bar charts retain this ranking*

The population of this supergroup (Fig. 8) tend to live in urban and semi-urban areas, predominantly concentrated around major cities of the Midlands, Yorkshire and the Humber, North West and North East (see Fig. 6). Residents tend to be female, living in single-person households, and to live in terraced housing or flats, with above-average representation in socially rented accommodation. They are more likely to live in income deprived households and experience fuel poverty. Residents are characterised by the lowest levels of educational attainment and internet engagement, provide high levels of unpaid care, suffer from poor health, and see the highest rates of prescribing dementia medications for more advanced conditions. The areas are characterised by the lowest median house prices, and crime rates tend to be higher.

#### Supergroup 2: Multicultural Central Urban Living

**Fig. 9:**
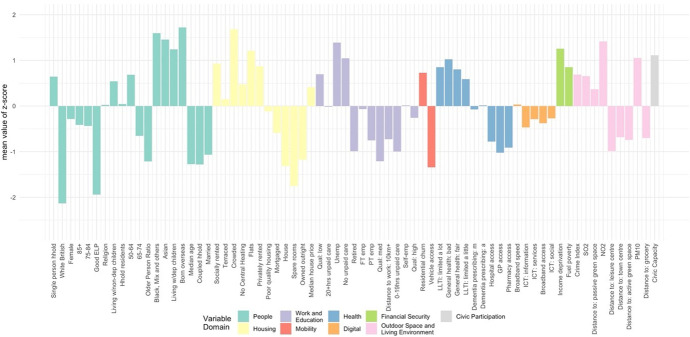
Profiles of supergroup “Multicultural Central Urban Living” (Mean value of z-score)

The population of this supergroup tend to live in city centres, with concentrations in major cities. This is the youngest and most ethnically diverse supergroup (Fig. 9), with higher-than-average proportions of residents born overseas, and of Asian, and Black, Mixed and Other ethnicities. There are also notably lower levels of English language proficiency. Residents are more likely to live in rented accommodation, particularly flats, and unlikely to have any spare rooms. The proportion of households without central heating is above the national average, and households in this supergroup are the most likely to experience income deprivation and fuel poverty. Nevertheless, median house prices are relatively high – typical of their central location. This supergroup has the lowest proportion of retirees, likely reflecting the younger age structure with a high proportion aged 50–64. However, employment rates are below average (though rates of self-employment are similar), and this supergroup has the highest rates of unemployment. The proportion of single-person households and living with children is higher than the national average, vehicle ownership is low, and residents tend to have relatively low levels of education. Though less likely to provide unpaid care, the likelihood of poor health and disability are also relatively high compared to the national average. Proximity to the city centre means distances to amenities and health services are amongst the shortest while the density of civic assets is the highest.

#### Supergroup 3: Rurban Comfortable Ageing

**Fig. 10:**
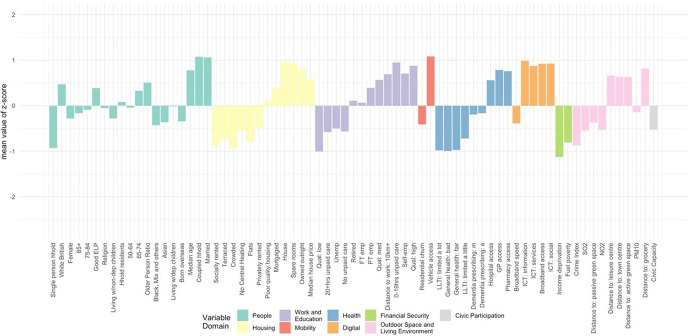
Profile of supergroup “Rurban Comfortable Ageing” (Mean value of z-scores)

The population of this supergroup predominantly live in rural, or rural–urban fringe areas. This is the largest and oldest supergroup (Fig. 10): it has the highest ratio of older people to younger people, and the highest median age reflects the concentration of older people in more rural areas. Residents are the most likely to be married and/or living as a couple. There is a high proportion of White British residents, with lower-than-average representation of ethnic minorities. This supergroup is most likely to own their properties outright and tend to live in detached/semi-detached housing or bungalows, and with spare rooms. This supergroup is the least likely to experience fuel poverty or to live in income deprived households. They tend to be in better health than the other supergroups and are most likely to provide between 0–19 h of unpaid care a week. They are relatively more likely to be either in self- or part-time employment and tend to have medium or higher levels of educational attainment. This is the most digitally engaged supergroup of older people. Their geography means that though they benefit from better air quality and lower crime rates, distance to services and amenities are amongst the highest. Accordingly, this supergroup is the most likely to have access to a vehicle.

#### Supergroup 4: Retired Fringe and Residential Stability

**Fig. 11:**
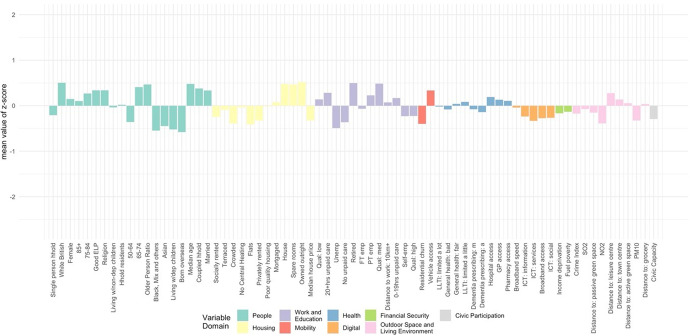
Profile of supergroup “Retired Fringe and Residential Stability” (Mean values of z-scores)

The population of this supergroup are concentrated in rural suburbs of smaller cities and towns, and coastal areas, particularly to the East. Residents of these areas are more likely to be between 65 and 84, with the highest proportion of retirees found across all clusters (Fig. 11). They are predominantly UK-born White British, are most likely to own their property outright and are likely to have spare rooms. This group represents a very stable population, with the lowest levels of residential mobility indicated across all clusters. However, the remaining characteristics in each domain are otherwise very close to the national average.

#### Supergroup 5: Cosmopolitan Comfort Ageing

**Fig. 12:**
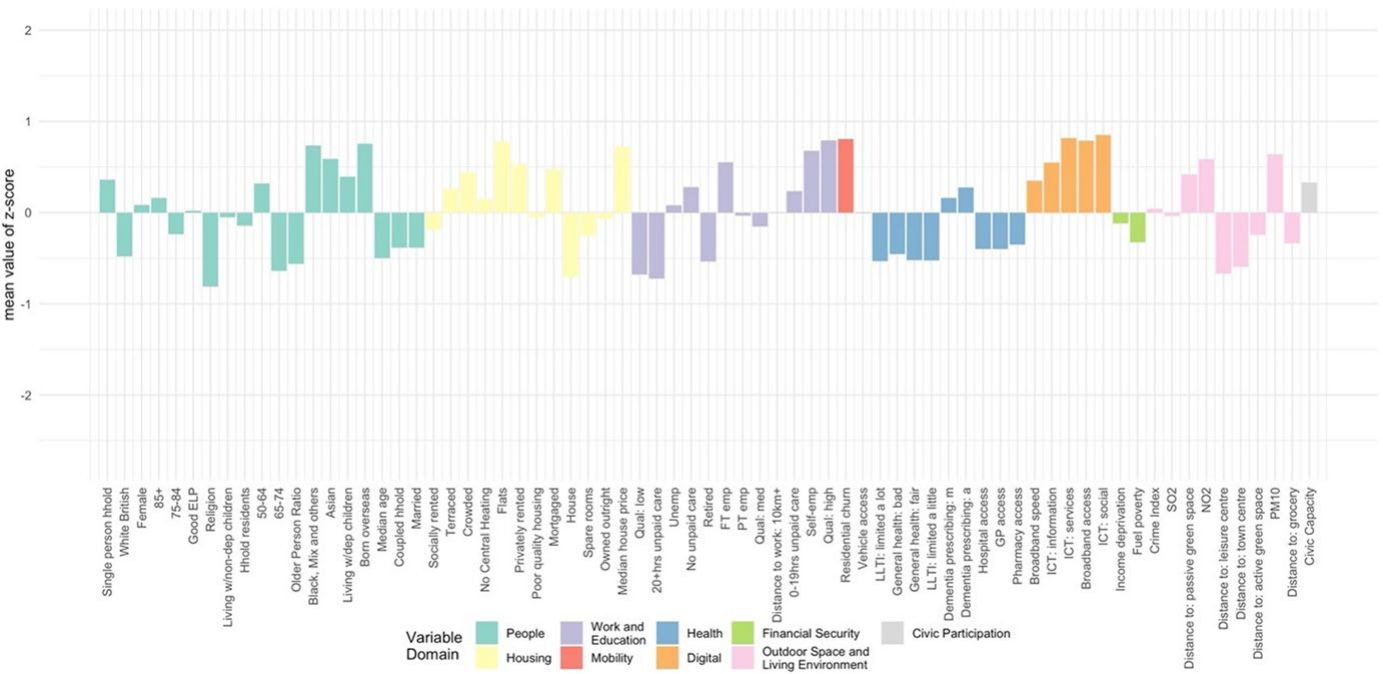
Profile of supergroup “Cosmopolitan Comfort Ageing” (Mean values of z-scores)

The population of this supergroup are concentrated in the periphery of major cities, or in the suburbs of towns, particularly around London and the South East. These areas are characterised by the highest median house price (mean value of £362,158.90). Residents are highly educated and likely to be either in full-time employment or self-employed (Fig. 12). They do not provide many hours of unpaid care, and are likely to live in property with a mortgage or shared ownership. There is also a higher proportion of people living in privately rented accommodation. Housing type tends towards terraced houses or flats, and there is an above-average rate of living in a crowded property. Though members of this group tend to have better health outcomes overall, there is a higher prescribing rate of dementia medications. People have access to high-speed broadband and like to engage with the internet, especially for shopping, banking and social use. These communities have the highest level of residential churn, and it is notable that there is also a relatively low ratio of older people to younger people in the local populations.

## Validation

A final stage of creating the bespoke geodemographics involved a ground-truthing validation exercise to understand better whether the clusters and the corresponding descriptions (names and pen portraits) were appropriately representing the real world. The framework was similar to the approach used in the work of Vickers and Rees (2011). A panel of peer reviewers were invited to validate the results by looking at the areas they know well, such as areas around their current and previous home addresses.

**Fig. 13:**
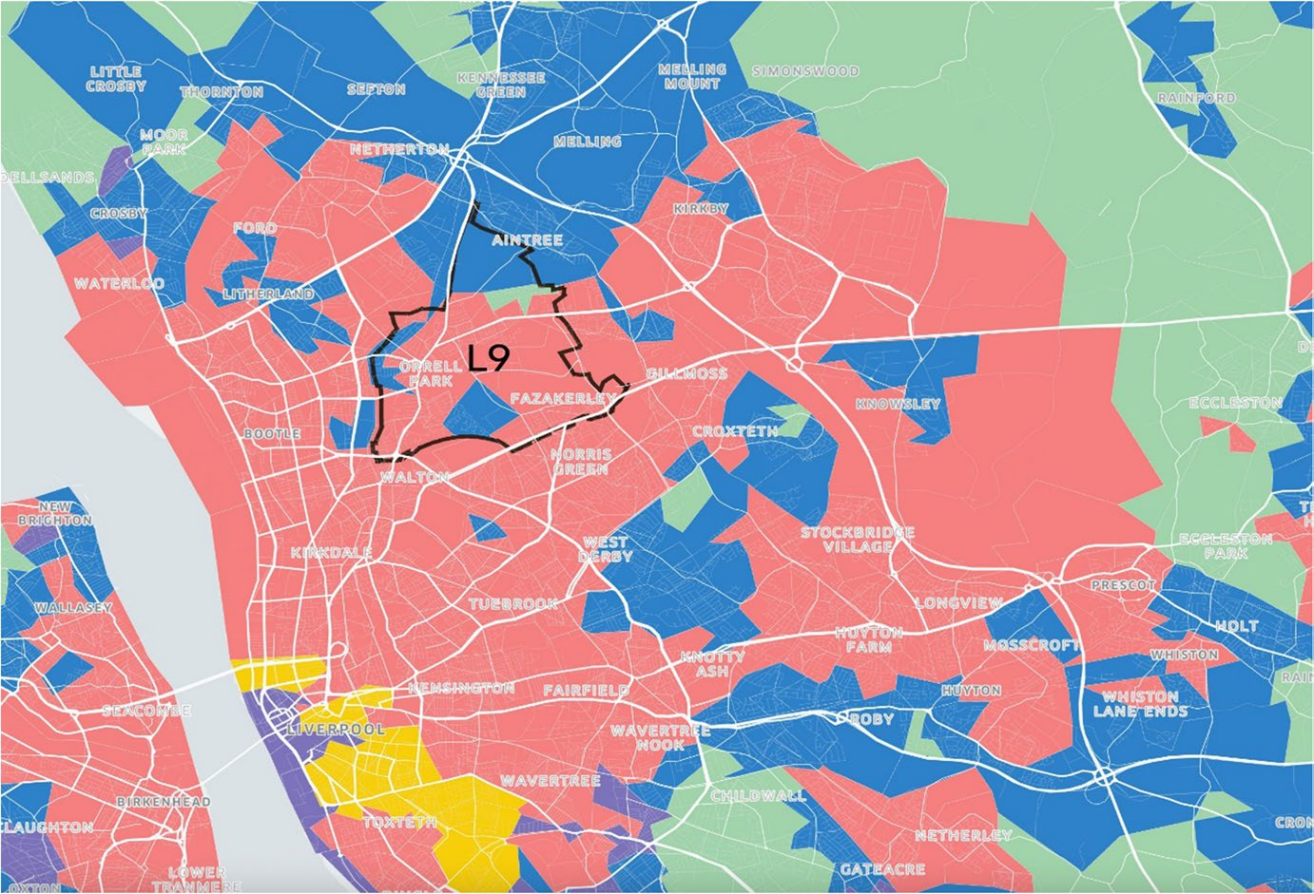
Map for ground-truthing the areas around postcode “L9”, which covers parts of North Liverpool

Participants were asked to provide up to 3 postcode districts, and maps were then created to show the postcode districts and the surrounding cities, towns or countryside (Vickers & Rees, 2011). Maps were then sent back to each participant respectively. Figure 13 is an example of the postcode district of “L9”, each supergroup is shaded with different colours on the map.

The correspondence between colours and supergroups were unknown to the participants. After reading the names and pen-portraits descriptions of the supergroups, participants would provide their answers to match each colour to the supergroups (see the examples in Table 4 with previous version of supergroup names). Respondents were asked to complete this “matching exercise” task based on their knowledge of the demographic, socioeconomic characteristics of older people and the environment in the local areas. They were also invited to provide further narrative comments, which were used to further inform evaluation and improvement of the AiPC.

**Table 4:**
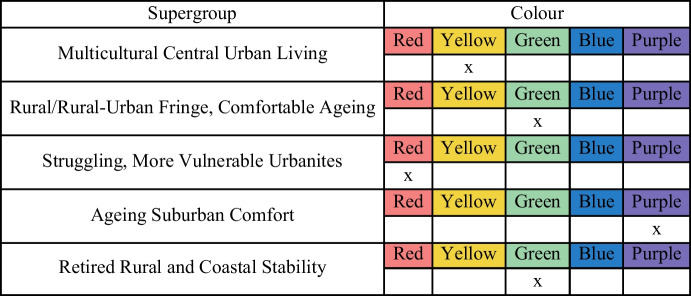
Ground-truthing form

**Fig. 14:**
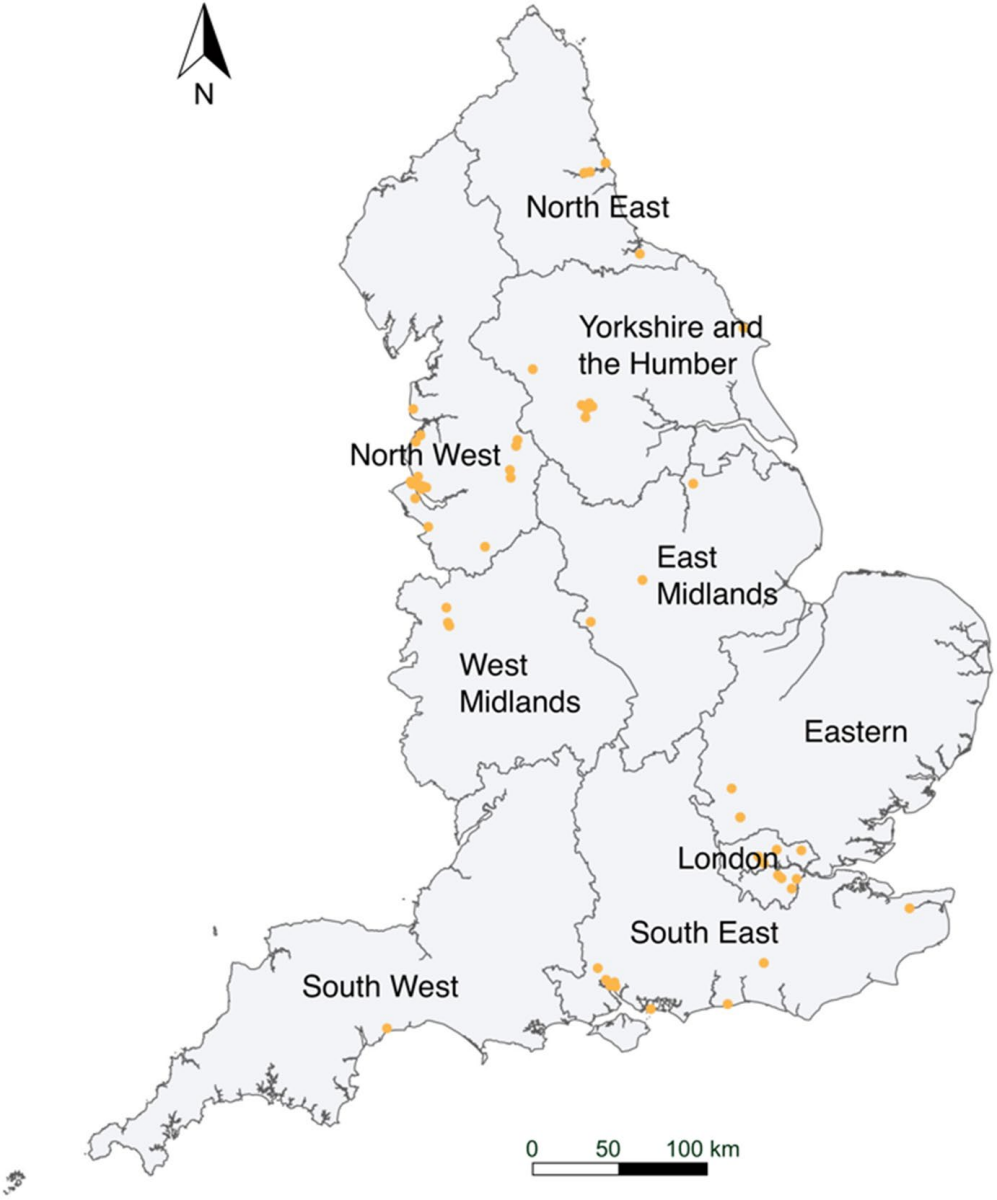
Ground-truthing sample locations (postcode districts)

A total of 56 non-duplicate locations (postcode districts) were provided by a panel of 25 peer reviewers, consisting of 9 PhD students and 16 external participants. The students were familiar with geography and urban planning, while the external participants were scholars and expert users of geodemographic classification. The responses were distributed across England, covering all regions in England (Fig. 14), with slightly more samples in the North West and London. The accuracy rates (%) for different supergroups are shown in Table 5.

**Table 5:** Accuracy rate (%) of different supergroups by different participants

Supergroup	External	Students	All
1: Struggling, More Vulnerable Urbanites	87.5	66.7	80
2: Multicultural Central Urban Living	75	50	66.7
3: Rurban Comfortable Ageing	78.6	55.6	69.6
4: Retired Fringe and Residential Stability	60	50	56.5
5: Cosmopolitan Comfort Ageing	66.7	87.5	73.9

The quantitative results (Table 5) and the qualitative feedback in the ground-truthing exercise were then discussed in a series of consultations with a panel of experts and end-users. Further changes were then made to finetune their names and descriptions.

For instance, the initial name of supergroup 4: “Retired Rural and Coastal Stability” had the lowest accuracy rate with only 56.5% of participants providing accurate response. Using geography in the cluster name was confusing to some respondents. Despite many LSOAs in this cluster located in coastal areas, a non-coastal and suburban distribution was also substantial. As such, the cluster name was altered to ‘Retired Fringe and Residential Stability’ to better reflect its data driven characteristics. Changes were also made to the initial names of clusters (supergroups) 3 and 5 and their descriptions, addressing issues raised by the respondents.

## Discussion and Conclusion

COVID19 illuminated an openly ageist narrative in public policy and the media, indicative of the long-standing marginalisation and homogenisation of older people. Old age is equated with dependency and frailty, with the ageing population framed as a burden on the national coffers. However, our experience of old age and of ageing is highly individualistic, itself determined by our wider socioeconomic characteristics, the places in which we live, and the places we have lived. Policy and service planning needs to better capture the social and spatial heterogeneity of the aged and ageing population if it is better meet the variable needs and potential opportunities an older and ageing population presents. To support that, a better more detailed understanding of the geography of older people is needed, moving beyond simplistic evaluations of ‘percentage aged 65 + ’ and ‘level of deprivation’. Building on existing advances in the creation of geodemographics, we present a bespoke geodemographic classification of older people in England. Developed in consultation with an expert advisory panel drawn from gerontologists, geriatricians, local authority policy makers, community workers, lay people and beyond, the domains have been critically interrogated to maximise the utility of this product for end users.

Our results show five distinctive clusters (Supergroups) and 13 nested subclusters (Groups) depicting distinctive features of the older population and their local environments. The AiPC is a multidimensional classification portraying differences and similarities between clusters and subclusters across nine key domains, distinguished by difference from the national average. The research output is, to the best of our knowledge, the first bespoke geodemographic classification of the older population for England, which utilises novel data sources including a consumer behaviour survey and NHS prescribing data, alongside more conventional sources (e.g. the Census). It should be noted that not all measures and data used as *k*-means inputs are readily available. Different models (spatial microsimulation, Xgboost) and novel secondary datasets (listed in Appendix 1) were applied to estimate digital engagement and obtain dementia prescription rates for ageing population at small area level. These measures are novel and provide robust metrics and new insights to enhanced the bespoke classification.

The process of building AiPC was grounded within a stakeholder community to maximise the utility of the research outputs and embed their design within a process of co-production. It provides new and valuable insights that can be implemented at both local and national contexts, in particular to improve service delivery and inform targeted policy interventions.

Though the methodology was rigorously designed, with all methodological decisions open to scrutiny and critique from our expert and stakeholder advisory board, the adopted approach is not without limitation. First, we make use of Census data from 2011 as the basis of the classification. As a cross-sectional snapshot of the population, these data are Census data are already outdated. However, at time of creation this was still the gold-standard available data and the insights gleaned remain of value. Second, surveys used to measure the digital engagement of the older population (e.g. British Population Survey) were not representative at small area level, therefore synthetic values had to be computed. Though this may introduce some discrepancies, the methods employed to compute the estimates are rigorous, tested methods used for such purposes. Third, NHS and Census data were used for estimating prescribing rates, while the two datasets are reported and updated in different time scales and frequency. The gaps between Census (2011) and prescribing (2015–2019) lead to higher uncertainty in the result, the migration of older people and changed age structure could all impact the accuracy of the estimated prescribing rates. This might be improved with the arrival of new Census. Finally, we were unable to capture some key attributes identified by our expert panel due to a lack of reliable data. One particular example related to the (perceived) safety of older people in their local environments. This was raised frequently as an important element, and is something to consider for future iterations.

Nevertheless, the combined critique and support of our advisory board, and the absence of such a classification to date, ensures the AiPC will have policy relevance and value. The AiPC is pertinent to the experiences of the current ageing population, offering clues to target routine policy development and service planning. Incorporating with small area estimation models, AiPC has the potential to be used to better understand older people’s loneliness, housing need, digital engagement gaps and more (Birkin & Clarke, 2012). It also provides insights into emergency preparation and local community resilience arguably missing from the Covid-19 response. Though local knowledge facilitated the rapid emergence of local mutual aid groups, wider regionally or nationally coordinated interventions and policy measures would have benefitted from a more nuanced understanding of the likely needs of local older populations, a group otherwise homogenised as vulnerable and in need. Beyond the immediacy of our current aged population, in incorporating features of the local environment pertinent to the experience of ageing, the classification will have continued resonance.

With the arrival of new Census data, it will be readily updateable as an open source product, further maximising its continued use in the face of a rapidly ageing population. The AiPC presented in this work could also be used for comparison as a snapshot of the past, providing a reference for identifying the changes in the geography and characteristics of older people in England. Future iterations must exploit the ever-growing availability of geo-referenced data to enhance the value of this classification, while end users should endeavour to illustrate where more age-disaggregated spatially referenced data is needed to further improve its performance.

## Figures and Tables

**Fig. 15:**
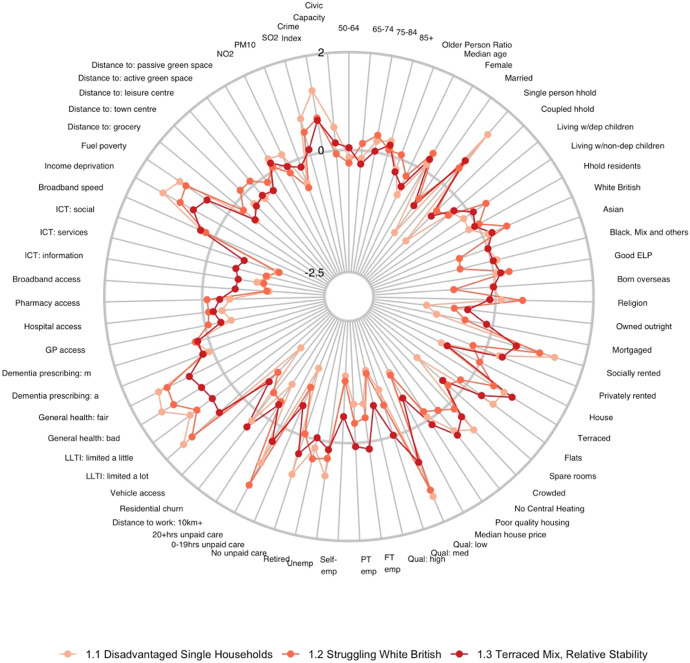
Radar plot of groups in supergroup “Struggling, More Vulnerable Urbanites”

**Fig. 16:**
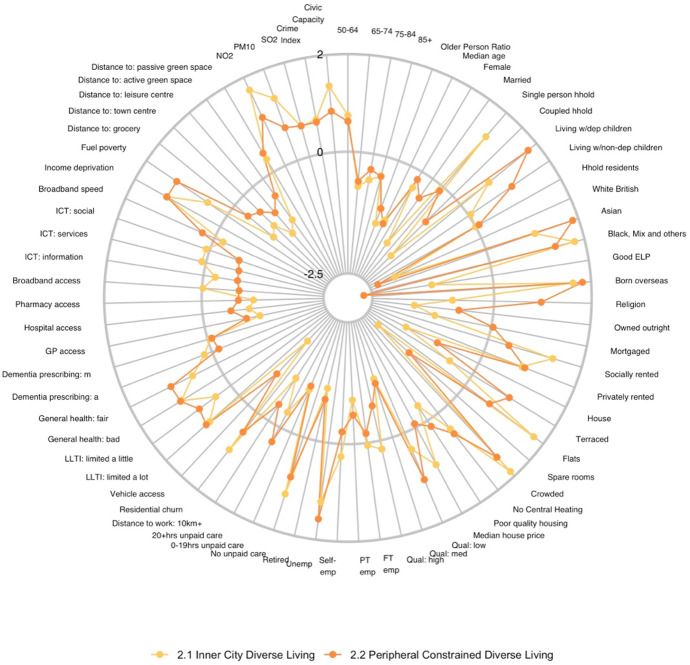
Radar plot of groups in supergroup “Multicultural Central Urban Living”

**Fig. 17:**
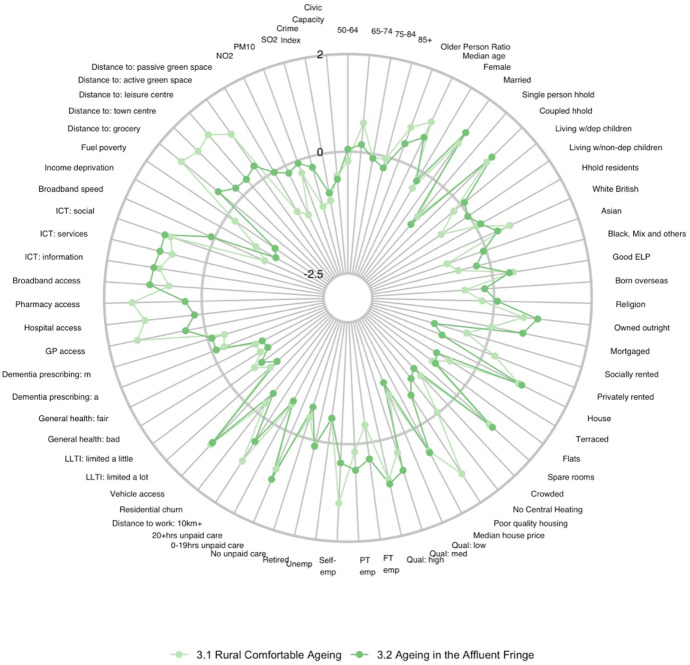
Radar plot of groups in supergroup “Rurban Comfortable Ageing”

**Fig. 18:**
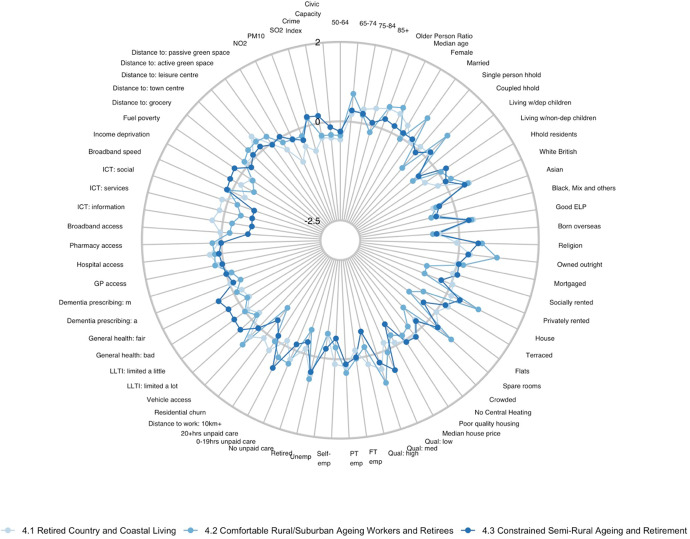
Radar plot of groups in supergroup “Retired Fringe and Residential Stability”

**Fig. 19:**
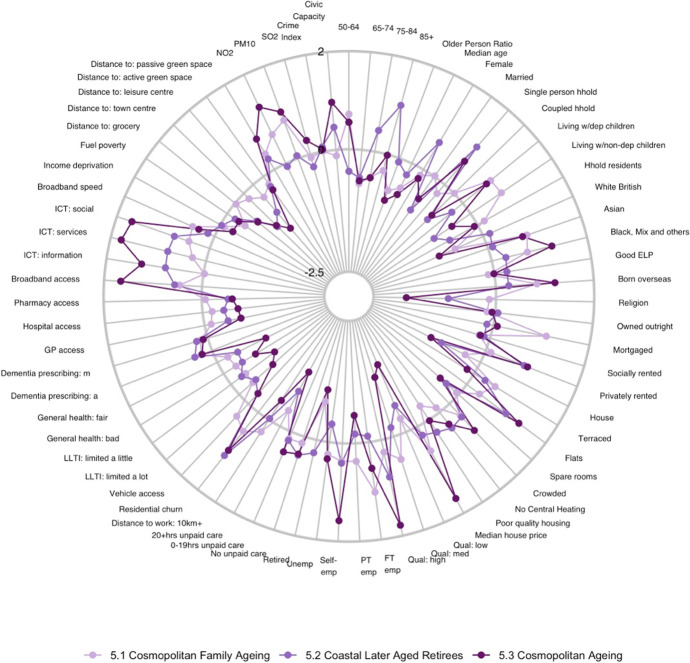
Radar plot of groups in supergroup “Retired Fringe and Residential Stability”

## Appendix 1

Tables 6, 7 and 8

**Table 6:** Domains and variables for AiPC

	Domain	Name	Description	Denominator
1	**People**	Age: 50-64 (%)	% of persons: aged 50 to 64	older people (age over 50)
2		Age: 65-74 (%)	% of persons: aged 65 to 74	older people (age over 50)
3		Age: 75-84 (%)	% of persons: aged 75 to 84	older people (age over 50)
4		Age: 85 and over (%)	% of persons: aged 85 and over	older people (age over 50)
5		Older Person Ratio	Population aged 65 and over, relative to population 18-64	people age 18-64
6		Median age	Median age (of all people)	all people
7		Female (%)	% of persons: female	older people (age over 50)
8		Marital status (%)	% of persons: married or in a registered civil partnership	older people (age over 50)
9		Single person household (%)	% of persons: living in single person household	older people (age over 50)
10		Coupled household (%)	% of persons: living in a couple	older people (age over 50)
11		Living with dependent children (%)	% of persons: living with dependent children	older people (age over 50)
12		Living with non- dependent children (%)	% of persons: living with non-dependent children	older people (age over 50)
13		Household residents (%)	% of persons: living in household (rather than communal establishment)	older people (age over 50)
14		White British (%)	% of persons: White British	older people (age over 50)
15		Asian (%)	% of persons: Asian/Asian British: Indian, Pakistani, Bangladeshi,Chinese and other Asian	older people (age over 50)
16		Black, mix and others (%)	% of persons: Black/African/Caribbean/Black British, Mixed or Other (including other white) ethnic groups	older people (age over 50)
17		Low English proficiency (%)	% of persons : cannot speak English or cannot speak English well	older people (age over 50)
18		Born overseas (%)	% of persons: non-UK born	older people (age over 50)
19		Religion (%)	% of persons: identified with a religion	older people (age over 50)
20	**Housing**	Owned outright (%)	% of persons: own the property outright	older people (age over 50)
21		with mortgage or shared ownership	% of persons: own the property with a mortgage, loan or shared ownership	older people (age over 50)
22		Socially rented (%)	% of persons: social renting	older people (age over 50)
23		Privately rented (%)	% of persons: private renting (includes living rent free)	older people (age over 50)
24		Detached, semi or bungalow housing (%)	% of persons (of all age): live in a detached or semi-detached house or bungalow	all households
25		Terraced housing (%)	% of persons (of all age): live in a terrace or end-terrace house	all households
26		Flats (%)	% of persons (of all age): live in a flat	all households
27		Spare rooms (%)	% of persons: household with 1 or more spare rooms	older people (age over 50)
28		Crowded (%)	% of persons: household with not enough rooms	older people (age over 50)
29		No central heating (%)	% of persons: household without central heating	older people (age over 50)
30		Poor quality housing (%)	% of social and private homes that fail to meet the decent home standard (related to hazards in the home, state of disrepair, modernisation, and thermal comfort)	all households
31		Median house price	Median house price	all households
32	**Work and Education**	Education: low (%)	% of persons: Other or No qualifications	older people (age over 50)
33		Education: medium (%)	% of persons: Level 1, 2 or Apprenticeship	older people (age over 50)
34		Education: high (%)	% of persons: Level 3, 4, or higher	older people (age over 50)
35		FT employed (%)	% of persons: full-time employed	older people (age over 50)
36		PT employed (%)	% of persons: part-time employed	older people (age over 50)
37		Self-employed (%)	% of persons: self-employed	older people (age over 50)
38		Unemployed (%)	% of persons: unemployed or economically inactive to look after home or family	older people (age over 50)
39		Retired (%)	% of persons: retired	older people (age over 50)
40		Care: 0 hour (%)	% of persons: provide more than 20 hours unpaid care a week	older people (age over 50)
41		Care: 1-19 Hours(%)	% of persons: provide more than 20 hours unpaid care a week	older people (age over 50)
42		Care: more than 20 hours (%)	% of persons: provide more than 20 hours unpaid care a week	older people (age over 50)
43		Travel to work: 10k+ (%)	% of persons: travel 10km or more for work	Economiclly active older (Age over 50) people
44	**Mobility**	Mobility (%)	% of households that have changed between the end of 2016 and the start of 2011, providing estimate of “churn” of the residential population	all households
45		Car access (%)	% of persons: car or van in household	older people (age over 50)
46	**Financial Security**	Income deprivation (%)	% of persons who living in a income deprived household (Income deprivation affecting older people index)	older people (age over 60)
47		Fuel poverty (%)	% of households in fuel poverty	all households
48	**Health**	LLTI: lot	Age Standardised Illness Ratio: Day-to-day activities limited a lot	older people (age over 50)
49		LLTI: little	Age Standardised Illness Ratio: Day-to-day activities limited a little	
50		General health: bad	Age Standardised Illness Ratio: Geaneral health of bad	older people (age over 50)
51		General health: fair	Age Standardised Illness Ratio: Geaneral health of fair	
52		Antidementia (A)	Prescribing rate of Acetylcholinesterase inhibitors (Antidementia) to each LSOA per person (50+) per year	older people (age over 50)
53		Antidementia (M)	Prescribing rate of Memantine (Antidementia) to each LSOA per person (50+) per year	older people (age over 50)
54		GP access	Average travel time to nearest GP by Public Transport and walking	all households
55		Hospital access	Average travel time to nearest Hospital by Public Transport and walking	all households
56		Pharmacy access	Average travel time to nearest Pharmacy by car	all households
57	**Digital**	Broadband access (%)	% of persons: broadband access at home	older people (age over 50)
58		ICT use: information (%)	% of persons (internet users aged 50+): use internet for information (hobbies, interests, services and products)	older people (age over 50) who use internet
59		ICT use: online shopping and banking (%)	% of persons (internet users aged 50+): use internet for online shopping and banking	older people (age over 50) who use internet
60		ICT use: social (%)	% of persons (internet users aged 50+): use internet for social networks and voice/video calls.	older people (age over 50) who use internet
61		Broadband speed	Average broadband download speed	older people (age over 50)
62	**Outdoor space and living environment**	Grocery	Average travel time to nearest Food Store by Public Transport and walking	all households
63		Town centre	Average travel time to nearest Town Centre by Public Transport and walking	all households
64		Leisure centre	Average road distance to nearest Leisure Centre	all households
65		Green space (active)	Average road distance to nearest green space	all households
66		Green space (passive)	Proportion of greenspace within a 900 m buffer (~15 minutes) from where people live	all households
67		Air Quality: NO2	Level of NO2	all households
68		Air Quality: PM10	Level of PM10	all households
69		Air Quality: SO2	Level of Sulphur Dioxide (SO2)	all households
70		Crime Index	composite index of crime rate (derived from IMD) in LSOA	all people
71	**Civic Participation**	Civic density	Number of civic assets within 1 km buffer of LSOA, divided by the number of people (of all age groups) in the LSOA	all people

**Table 7:** List of secondary datasets (except Census) and the corresponding variables

Dataset	Variable	Description
British Population Survey	Broadband access (%)	% of persons: broadband access at home
	ICT use: information (%)	% of persons (internet users aged 50+): use internet for information (hobbies, interests, services and products)
	ICT use: online shopping and banking (%)	% of persons (internet users aged 50+): use internet for online shopping and banking
	ICT use: social (%)	% of persons (internet users aged 50+): use internet for social networks and voice/video calls.
CDRC Broadband Speed Data	Broadband speed	Average broadband download speed
Journey time statistics	Grocery	Average travel time to nearest Food Store by Public Transport and walking
	Town centre	Average travel time to nearest Town Centre by Public Transport and walking
	GP access	Average travel time to nearest GP by Public Transport and walking
	Hospital access	Average travel time to nearest Hospital by Public Transport and walking
	Pharmacy access	Average travel time to nearest Pharmacy by car
Access to Healthy Assets & Hazards Data	Leisure centre	Average road distance to nearest Leisure Centre
	Green space (active)	Average road distance to nearest green space
	Green space (passive)	Proportion of greenspace within a 900 m buffer (~15 minutes) from where people live
	Air Quality: NO2	Level of NO2
	Air Quality: PM10	Level of PM10
	Air Quality: SO2	Level of Sulphur Dioxide (SO2)
English Indices of multiple deprivation (2019)	Crime Index	composite index of crime rate (derived from IMD) in LSOA
	Income deprivation (%)	% of persons who living in a income deprived household (Income deprivation affecting older people index)
	Poor quality housing (%)	% of social and private homes that fail to meet the decent home standard (related to hazards in the home, state of disrepair, modernisation, and thermal comfort)
Ordnance Survey Point of Interests data	Civic density	Number of civic assets within 1 km buffer of LSOA, divided by the number of people (of all age groups) in the LSOA
NHS English Prescribing data and registered patients data	Antidementia (A)	Prescribing rate of Acetylcholinesterase inhibitors (Antidementia) to each LSOA per person (50+) per year
	Antidementia (M)	Prescribing rate of Memantine (Antidementia) to each LSOA per person (50+) per year
Low Income Low Energy Efficiency (LILEE) data in England	Fuel poverty (%)	% of households in fuel poverty
CDRC Residential Mobility Index	Mobility (%)	% of households that have changed between the end of 2016 and the start of 2011, providing estimate of “churn” of the residential population
Median house prices by lower layer super output area: HPSSA dataset 46	Median house price	Median house price

**Table 8:** List of secondary datasets (except Census) and their source/authors (links can be accessed on 1 July 2022)

Dataset	Source/Authors	Link and reference
British Population Survey	Consumer Data Research Centre	https://data.cdrc.ac.uk/dataset/british-population-survey
CDRC Broadband Speed Data	Consumer Data Research Centre	https://data.cdrc.ac.uk/dataset/broadband-speed
Journey time statistics	Department for Transport	https://www.gov.uk/government/collections/journey-time-statistics
Access to Healthy Assets & Hazards Data	Green et al., (2018)	https://www.cdrc.ac.uk/new-update-access-to-healthy-assets-and-hazards-ahah-data-resource/ Green, M. A., Daras, K., Davies, A., Barr, B., & Singleton, A. (2018). Developing an openly accessible multi-dimensional small area index of ‘Access to Healthy Assets and Hazards’ for Great Britain, 2016. *Health & Place*, *54*, 11–19.
English Indices of multiple deprivation (2019)	Ministry of Housing, Communities & Local Government	https://www.gov.uk/government/statistics/english-indices-of-deprivation-2019
Ordnance Survey Point of Interests data	Ordnance Survey	https://www.ordnancesurvey.co.uk/business-government/tools-support/points-of-interest-support
NHS English Prescribing data and registered patients data	NHS Digital	https://www.nhsbsa.nhs.uk/prescription-data/prescribing-data/english-prescribing-data-epd https://digital.nhs.uk/data-and-information/publications/statistical/patients-registered-at-a-gp-practice
Low Income Low Energy Efficiency (LILEE) data in England	Department for Business, Energy & Industrial Strategy	https://www.gov.uk/government/collections/fuel-poverty-statistics
CDRC Residential Mobility Index	Consumer Data Research Centre	https://data.cdrc.ac.uk/dataset/cdrc-residential-mobility-index
Median house prices by lower layer super output area: HPSSA dataset 46	Office for National Statistics	https://www.ons.gov.uk/peoplepopulationandcommunity/housing/datasets/medianpricepaidbylowerlayersuperoutputareahpssadataset46

## Appendix 2

### AiPC groups characteristics and Pen Portraits

#### Groups in Supergroup 1 “Struggling, More Vulnerable Urbanites”

There are three groups in the second tier of supergroup “Struggling, More Vulnerable Urbanites”. Figure 15 shows the radar plot of the groups across the variables, and their characteristics are summarised below.

##### Group 1.1 Disadvantaged Single Households

Shares a similar age-structure to parent group, with the highest proportion of people aged 85 + despite the lowest median age at 34.68. A relatively more diverse population than parent group but with similarly high proportions in single-person households and socially rented accommodation. Households are less likely to have spare rooms and most likely to experience income deprivation and fuel poverty. This group are the least educated and least likely to engage digitally, but with the highest capacity for civic engagement.

##### Group 1.2 Struggling White British

The median age is slightly higher than parent group (37.75 compared to 36.25) and with a slightly higher proportion aged 65–74. Higher proportion of White British than the parent group, and relatively more likely to identify with a religion. Though below the national average, they are more likely to live in detached/semi-detached houses or bungalows than parent group and, overall, more likely to provide unpaid care. As this group tend to locate at semi-urban (urban fringe) areas, travel distances to local amenities and health services tends to be greater than the other two subgroups.

##### Group 1.3 Terraced Mix, Relative Stability

This group is slightly younger than the parent group, with a slightly higher proportion of aged 50–64. They are characterised by higher levels of educational attainment, employment and digital engagement than the parent group, and are more likely to own their own home, enjoy more financial security and are less likely to be in poor health. Though living in strained circumstances compared to the national average, this subgroup are relatively better off than the two sister clusters.

#### Groups in Supergroup 2 “Multicultural Central Urban Living”

There are two groups in the second tier of supergroup “Multicultural Central Urban Living”. Figure 16 shows the radar plot of the groups across the variables, and their characteristics are summarised below.

##### Group 2.1 Inner City Diverse Living

This group tend to concentrate within inner city areas, with higher median house prices than the parent group. Residents are far more likely to live in socially rented accommodation, concentrating in flats and less likely to have spare rooms. It is an ethnically diverse cluster, but as compared to the parent group with relatively lower proportions of Asian ethnicities and higher proportions of Black, Mixed and Other ethnicities. Though residents are more likely to live in income deprived households, they have a lower risk of fuel poverty. Given proximity to the city centre, the low levels of vehicle ownership and short distances to services and amenities is unsurprising.

##### Group 2.2 Peripheral Constrained Diverse Living

This group concentrates on the periphery of town and city centres, with notably lower median house prices than the parent group. Residents are more likely to be of Asian ethnicity and to identify with a religion than in any other cluster reported, and residents have the lowest levels of English language proficiency. They are more likely to be living as a couple or married than the parent group, residing in terraces, detached/semi-detached housing, or bungalows, either mortgaged or owned outright. However, housing quality is poorer than the parent group, and households are at greater risk of fuel poverty. Rates of self-employment are lower while there are a higher proportion of retirees.

#### Groups in Supergroup 3 “Rurban Comfortable Ageing”

There are two groups in the second tier of supergroup “Rurban Comfortable Ageing”. Figure 17 shows the radar plot of the groups across the variables, and their characteristics are summarised below.

##### Group 3.1 Rural Comfortable Ageing

This group concentrate in rural areas, with the highest travel distances to healthcare facilities, services and amenities. This group have less financial security than the parent group, are more likely to live in privately or socially rented accommodation and less likely to have a mortgage. Though this group tend to have central heating, the housing quality is relatively poor. Of those working, more are in self-employment than the parent group. Digital engagement is slightly lower than in the parent group, with notably lower proportions of people with broadband access at home and a lower broadband speed available.

##### Group 3.2 Ageing in the Affluent Fringe

Members in this groups tend to reside in more affluent areas on the urban–rural fringe, and are less likely to experience financial hardship than the parent group. Housing tends to be of good quality with detached/semi-detached housing and bungalows dominating the dwelling types. Higher proportions are married, living as a couple, and/or with children. This group has the highest level of digital engagement across different online activities of all clusters, and experience relatively better health than the parent group.

#### Groups in Supergroup 4 “Retired Fringe and Residential Stability”

There are two groups in the second tier of supergroup “Retired Fringe and Residential Stability”. Figure 18 shows the radar plot of the groups across the variables, and their characteristics are summarised below.

##### Group 4.1 Retired Country and Coastal Living

Relative to the parent supergroup, there are more people aged 75–84 and 85 and over in this group, and residents have a slightly higher tendency to higher levels of educational attainment, and being self-employed. Residents also have a higher probability of living in communal establishments (rather than a household), and are less likely to identify with a religion. Despite notably lower broadband speeds, levels of digital engagement are relatively high. As many areas in this group are in coastal areas and further from larger urban areas, members of this group benefit from significantly better air quality, but also have high levels of vehicle access and longer distance commutes.

##### Group 4.2 Comfortable Rural/Suburban Ageing Workers and Retirees

A slightly younger age-structure relative to the parent group, with a higher proportion aged 50–64 and 65–74. Residents are likely to be married or living as a couple, with a low proportion of single person households. They tend to own their property which is more likely to be detached /semi-detached housing or a bungalow, and less likely to be overcrowded. Of those working, there are slightly more people in part-time employment. These areas are very stable, experiencing the lowest level of residential churn within this supergroup.

##### Group 4.3 Constrained Semi-Rural Ageing and Retirement

This group may be living in relatively more constrained circumstances than the parent group: they have relatively lower levels of educational attainment, are more likely to be unemployed, provide more than 20 h of unpaid care a week, and tend to have poorer health outcomes. This group are much less likely to engage with the internet and more likely to be affected by household income deprivation and fuel poverty. The areas experience higher crime rates and the median house price is lower.

#### Groups in Supergroup 5 “Cosmopolitan Comfort Ageing”

There are two groups in the second tier of supergroup “Cosmopolitan Comfort Ageing”. Figure 19 shows the radar plot of the groups across the variables, and their characteristics are summarised below.

##### Group 5.1 Cosmopolitan Family Ageing

This group has slightly higher proportion of ages 50–64 than the parent group with people more likely to live in mortgaged properties, particularly detached/semidetached housing. A higher proportion of this group have lower levels of educational attainment relative to the parent group, and there is also greater representation of people born overseas and from Asian ethnicities. Single person households are relatively less common and residents are more likely to be living with children, married and/or living as a couple.

##### Group 5.2 Coastal Later Aged Retirees

Compared to the parent group, there are notably higher proportions of White British residents in these areas, with much lower representation of ethnic minorities or residents born overseas. Residents are also notably older, both than the average national population for ages 85 and over, and the parent group for 75 and over. They are likely to be female, living in single-person households and retired. However, there is also a higher proportion living in communal establishments than many other areas. These areas tend to have relatively high levels of residential mobility.

##### Group 5.3 Cosmopolitan Ageing

Residents are highly educated, less likely to be retired, and slightly younger than the parent group. Ethnic minorities, particularly Black, Mixed and Other ethnicities and those born overseas are more highly represented in this group. Residents are more likely to live in crowded households, particularly flats. These areas affluent suburbs of cities. The median house price in these areas is very high, but the centrality means residents benefit from good proximity to a range of amenities and civic assets.

## Footnotes

1 https://www.nhsbsa.nhs.uk/prescription-data/prescribing-data/english-prescribing-data-epd.

2 https://digital.nhs.uk/data-and-information/publications/statistical/patients-registered-at-a-gp-practice.
